# Chemical Vapor Deposition of Photocatalyst Nanoparticles on PVDF Membranes for Advanced Oxidation Processes

**DOI:** 10.3390/membranes8030035

**Published:** 2018-06-21

**Authors:** Giovanni De Filpo, Elvira Pantuso, Katia Armentano, Patrizia Formoso, Gianluca Di Profio, Teresa Poerio, Enrica Fontananova, Carmen Meringolo, Alexander I. Mashin, Fiore P. Nicoletta

**Affiliations:** 1 Department of Chemistry and Chemical Technologies, University of Calabria, Via P. Bucci Cubo 15/C, 87036 Rende (CS), Italy; giovanni.defilpo@unical.it; 2 Department of Pharmacy, Health and Nutritional Sciences, University of Calabria, Via P. Bucci Edificio Polifunzionale, 87036 Rende (CS), Italy; elvirapnt.ep@gmail.com (E.P.); katiarmentano@hotmail.it (K.A.); patrizia.formoso@unical.it (P.F.); 3 National Research Council of Italy (CNR)—Institute on Membrane Technology (ITM), Via P. Bucci Cubo 17/C, 87036 Rende (CS), Italy; g.diprofio@itm.cnr.it (G.D.P.); t.poerio@itm.cnr.it (T.P.); e.fontananova@itm.cnr.it (E.F.); c.meringolo@itm.cnr.it (C.M.); 4 Applied Physics & Microelectronics, Lobachevsky State University of Nizhni Novgorod, 603950 Nizhni Novgorod, Russia; mashin@unn.ru

**Keywords:** chemical vapor deposition, polyvinylidene difluoride, titanium dioxide, zinc oxide, photocatalysis

## Abstract

The chemical binding of photocatalytic materials, such as TiO_2_ and ZnO nanoparticles, onto porous polymer membranes requires a series of chemical reactions and long purification processes, which often result in small amounts of trapped nanoparticles with reduced photocatalytic activity. In this work, a chemical vapor deposition technique was investigated in order to allow the nucleation and growth of ZnO and TiO_2_ nanoparticles onto polyvinylidene difluoride (PVDF) porous membranes for application in advanced oxidation processes. The thickness of obtained surface coatings by sputtered nanoparticles was found to depend on process conditions. The photocatalytic efficiency of sputtered membranes was tested against both a model drug and a model organic pollutant in a small continuous flow reactor.

## 1. Introduction

Thin films are material layers that have thicknesses varying from tens of nanometers to a few micrometers [1]. They are generally obtained by deposition processes on the surface of given substrates. The thin film growth is generally a two-step process where an initial random nucleation step is followed by an ordered growth. Nucleation and growth—and consequently the film structure—depend on the substrate chemistry (surface composition and structure), the method used [2], and deposition conditions [3].

Thin film deposition methods are classified in solid, liquid, and gas phase deposition methods according to the physical state of the deposited material. A further classification of gas deposition methods distinguishes chemical vapor deposition (CVD) and physical vapor deposition (PVD) processes. Both methods involve the deposition of atoms or molecules carried in their vapor phase onto the substrate surface. In a CVD process, the deposited (target) material reacts chemically with the substrate, while in PVD processes, the deposited molecules and substrate are still distinct [4].

Separation, concentration, and purification processes present some challenges for chemical industries. Efficient separation and purification are also important for food and pharmaceutical plants in order to guarantee high-quality water after removal of toxic components from industrial wastewater. Pharmaceutical active compounds can be considered as hazardous substances because of their potential threat to health and the environment. They are also considered an emerging pollutant due to the failure of classical treatments (such as filtration, adsorption, bio-oxidation, sedimentation, coagulation, chlorination, and UV-irradiation) to effectively remove them [5,6,7]. In addition to common chemical pollutants, over 80 pharmaceutical active compounds have been detected in wastewater effluents and surface water across the world [8], with concentration ranging from few ng L^−1^ to several g L^−1^. The most important sources of pharmaceutical active compounds are incorrect disposal of unused drugs and effluents of wastewater treatment plants (including pharmaceutical industries, hospital wastewater, aqua-farming, and cattle-breeding) [9,10,11]. While the measured concentrations can result in water that is low or below drinking water guidelines and health criteria [12], their continuous accumulation in aquatic environment can also represent a real hazard.

More recently, polymer membranes have been used as innovative separation materials [13]. A membrane can be defined as “a barrier that separates and/or contacts two different regions and controls the exchange of matter and energy between the regions” [14]. Today, membranes are efficiently used for water desalinization, wastewater purification, recovery of valuable constituents from production waste, gas separation in petrochemical processes, concentration and purification in food and drug applications, artificial organs and therapeutic systems, energy conversion, and storage systems [15,16,17]. In addition to their technical simplicity and energy efficiency, membrane processes can be easily upscaled from batchwise treatment of small quantities to large-scale continuous operations.

An advanced oxidation process (AOP) is a simple technique that allows an efficient degradation of organic pollutants generally found in wastewater. In an AOP, organic pollutants are mineralized by the generation of highly reactive hydroxyl radicals [18]. Among the advanced treatment technologies, UV photocatalysis by nanoparticles (e.g., ZnO and TiO_2_), has attracted great interest in recent years [19].

**Figure 1:**
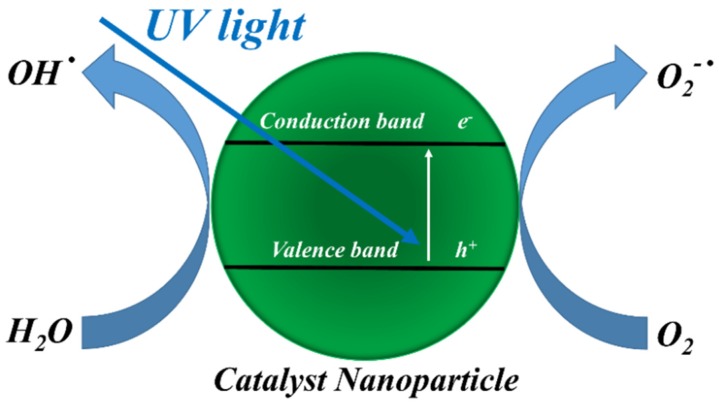
Schematization of the photoactivity of a catalyst nanoparticle (*CNp*).

In a photooxidation process (Figure 1), electrons are promoted from valence band to conduction band—resulting in the formation of electron-hole pairs—when the catalyst nanoparticle (*CNp*) is irradiated by UV light with energy intensity larger than the characteristic band gap (3.37 eV for ZnO and 3.2 eV for TiO_2_, respectively) [20,21].

Both electrons and holes can move to the semiconductor surface and produce radicals, which can oxidize organic compounds (*OC*), whereas electrons can reduce them, according to the reactions reported [19,22]. The degradation of organic compounds (*OC*) by photocatalyst nanoparticles (*CNp*) is shown below.

$$\begin{array}{l} CNp+h\nu \to e_{cb}^{-}+h_{vb}^{+}\\ O_{2}+e_{cb}^{-}\to O_{2}^{-•}\\ H_{2}O+h_{vb}^{+}\to OH^{•}+H^{+}\\ O_{2}^{-•}+H_{2}O\to H_{2}O_{2}\to 2OH^{•}\\ OH^{•}+OC\to OC_{ox}\\ OC+e_{cb}^{-}\to OC_{red} \end{array}$$

ZnO and TiO_2_ are the most commonly used photocatalysts due to their redox ability, chemical stability, reduced toxicity towards the environment and health, and low cost [23]. In addition to the mineralization of organic compounds, the reactive redox species—such as hydroxyl radicals ($OH^{•}$), superoxide anions ($O_{2}^{-•}$), and hydrogen peroxide molecules (H_2_O_2_) generated by UV irradiation—can damage the cell membrane of microorganisms [24] and kill bacteria, viruses, fungi, and algae [25], thus conferring long-term antibacterial and antifungal properties [26,27,28] to photocatalysts.

More recently, submerged membranes have been integrated by semiconductor photocatalysts in order to photomineralize membrane fouling [29,30], thereby reducing cleaning and maintenance costs. In particular, Ho et al. obtained an enhancement in the filtration flux of a submerged membrane reactor by integration of photooxidation process and membrane filtration [31], while Mendez-Arriaga et al. [32] studied the TiO_2_ photocatalytic degradation of pharmaceutical compounds such as diclofenac, naproxen, and ibuprofen.

The combination of membrane separation and advanced oxidation processes is an emerging technology for the complete removal of pollutants because each technique complements the advantages of the other. In particular, the AOP eliminates membrane fouling and allows the remediation of the concentrate while, at the same time, the membrane process filters the feed and concentrates pollutants to an optimal level for AOP [33,34,35].

Nevertheless, the chemical binding of photocatalysts onto porous polymer membranes requires a series of chemical reactions and long cleaning processes, which often result in small amounts of trapped nanoparticles with reduced photocatalytic activity. In addition, some polymers, such as polytetrafluoroethylene and polyvinylidene fluoride, are hardly functionalizable in order to trap photocatalyst molecules.

In this work, a chemical vapor deposition process was investigated in order to allow the nucleation and growth of ZnO and TiO_2_ photocatalytic nanoparticles onto polyvinylidene difluoride (PVDF) porous membranes for applications in AOP.

The purpose of this work was the coupling of filtration properties of polymer membranes with the photocatalytic activity of ZnO and TiO_2_ nanoparticles nucleated and grown on PVDF porous membranes using the CVD technique. The substrates used were membrane disks in PVDF, which is a thermoplastic material characterized by high strength and nontoxicity and, consequently, widely used in membrane processes and food applications. Moreover, PVDF is characterized by high chemical and UV stability, which renders this material particularly interesting for photocatalytic applications.

**Figure 2:**
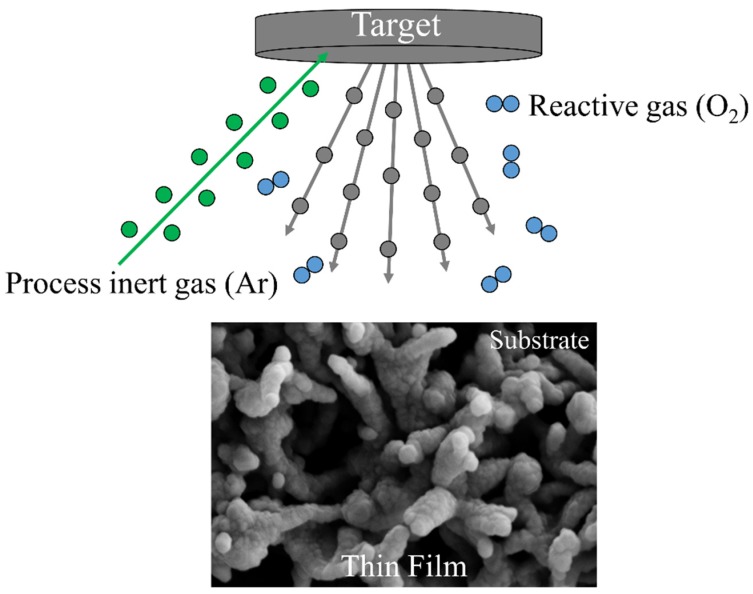
Schematization of the chemical vapor deposition (CVD) technique. The substrate is exposed to one or more volatile precursors, which react on the substrate surface to produce the desired thin film.

CVD is a well-known chemical process for the production of high-purity, high-performance solid thin films. In a typical CVD process, the substrate is exposed to one or more volatile precursors, which react on the substrate surface to produce the desired layer (Figure 2).

The photocatalytic efficiency of sputtered membranes was tested against a model drug (diclofenac sodium) and a model pollutant (methylene blue) in a small continuous flow reactor.

## 2. Materials and Methods

The substrates used were PVDF membrane disks with a diameter of 47 mm, a porosity of 70%, and a mean pore size of 0.10 μm (Durapore ©, Merck KGaA, Darmstadt, Germany). Prior to use, membranes were washed in methanol (Sigma-Aldrich, Milan, Italy) by an ultrasonic bath (model M1800H-E, Bransonic, Danbury, CT, USA). The deposition of nanostructured photocatalysts on PVDF membranes was obtained by sputtering of appropriate targets by process inert gas ions (argon) in a Edwards AUTO-306 sputtering system (Edwards, Burgess Hill, UK).

ZnO was deposited on PVDF membranes by Argon (purity 99.999%) sputtering of a ZnO target (purity 99.99%, Goodfellow Cambridge Ltd., Huntingdon, England). The deposition of nanostructured TiO_2_ was obtained by reactive sputtering using a Ti target (purity 99.999%, Goodfellow Cambridge Ltd., Huntingdon, England) in presence of a gaseous mixture of argon and oxygen (purity 99.999%, pressure of gas mixture: p(Ar) = 2.8 × 10^−3^ mbar and p(O_2_) = 1.2 × 10^−3^ mbar, p(Ar)/p(O_2_) = 2.3). The reactive gas mixture reacts with the substrate and sputtered atoms, forming a thin film of desired compound onto the substrate. The particular pressure ratio between Ar and O_2_ was chosen in order to form the anatase polymorph of TiO_2_ [36], which is more photoactive than rutile polymorph [37].

A microRaman spectrometer (Labram, Horiba Jobin Yvon) equipped with an Olympus microscope and interfaced to a color camera was used to confirm the presence of TiO_2_ anatase thin layers. The Raman spectra were collected through a 100× objective using a He–Ne laser (emission wavelength at 632.8 nm, power 5 mW).

Membrane and nanoparticle morphology was investigated by scanning electron microscopy (SEM). Observations were performed on membrane top surfaces coated with a thin gold or graphite film by a Leica LEO 420 (Leica Microsystems, Cambridge, England) or a Quanta 200 (FEI/Philips, Eindhoven, Netherlands) scanning electron microscope equipped with a backscatter electron detector. Energy-dispersive X-ray (EDX) maps were obtained with a Phenom ProX SEM (Phenom-World, Eindhoven, The Netherlands). Transmission electron microscope (TEM) images were collected with a JEM 1400 Plus transmission electron microscope operating at 100 kV (Jeol, Akishima, Tokyo, Japan). The shape and size of nanoparticles was obtained by software analysis of TEM pictures. The number of measured nanoparticles—taken from different pictures of the same sample—was at least 100, and their size was evaluated with an image software (Motic Images Plus 2.0, MoticEurope S.L.U., Barcelona, Spain).

Static contact angles to pure water were measured with a CAM 200 contact angle meter (KSV Instruments LTD, Helsinki, Finland) at 25 °C. A drop (2 µL) of water was put onto the sample surface by a microsyringe, and measurements were carried out by setting the tangents on both visible edges of the droplet on five different positions of each sample and calculating the average value of the measurements.

The permeation tests were carried out with distilled water using a tangential flow filtration cell having an active area of 14.5 cm^2^. The feed solution (at 25 ± 1 °C) was pumped parallel to the membrane surface by a gear pump at the transmembrane pressure of 0.4 bar. The feed flow rate was 250 mL min^−1^. Permeate samples were collected every 5 min in order to determine the transmembrane flux, *J*, defined as:

(1) $$J=\frac{V_{p}}{A\;\Delta t}$$

where *V_p_* is the permeate volume passed through the membrane in the fixed time interval Δ*t* and *A* is the effective membrane area. The photoactivity of ZnO and TiO_2_ layers was tested in a small continuous plant where either a diclofenac sodium (9.3 × 10^−5^ M, Sigma Aldrich, Milan, Italy) or methylene blue (1.3 × 10^−5^ M, Sigma Aldrich, Milan, Italy) water solution was recirculated by a peristaltic system through a round cell, which was equipped with a quartz window to allow UV irradiation and divided into two compartments by membrane [38]. The sputtered side of membranes was exposed to the UV light from a medium-high pressure mercury vapor lamp (ZS lamp, Helios Italquartz, Italy) with an irradiance of 6 W m^−2^. At the cell exit, the solution passed through a quartz flow cuvette placed inside a spectrophotometer able to read the absorbance value at either 275 nm or 665 nm, which are the wavelength of maximum absorption of diclofenac sodium and methylene blue, respectively. The absorbance was measured at regular intervals of 5 or 15 min.

## 3. Results and Discussion

The quality of the obtained thin film is strongly dependent on the process parameters. In particular, the sputtering yield (*Y*) is defined as the number of sputtered atoms per impinging ion. Consequently, a higher yield results in a higher sputtering deposition rate. The sputtering yield depends on several parameters [39], including the energy of the incident ions, the masses of the ions and target atoms, the binding energy of atoms in the solid, and the incident angle of ions. The sputtering yield can be expressed as:

(2) $$\;Y=\alpha \frac{Mm\;E_{m}}{{\left(M+m\right)}^{2}U_{M}}$$

where *m* and *M* are the mass of the bombing ion and target atom, respectively, *E_m_* is the kinetic energy of bombing ion, and *U_m_* is the bonding energy of target metal. *α* takes into account the incident angle of ions.

It is important to recall that magnetic field strength, CVD chamber volume, power density, gas composition and pressure are other factors that can affect yield values [40]. In addition, the film deposition rate decreases for increasing target-substrate distances. Therefore, under the chosen sputtering process parameters (obtained starting from values based on the previous works [41,42] using the same sputtering source), an optimal deposition rate for ZnO and TiO_2_ of about 2 and 1 nm min^−1^, respectively, was gained. The sputtering time used for the ZnO target was twice that of the Ti one in order to have similar layer thicknesses.

**Figure 3:**
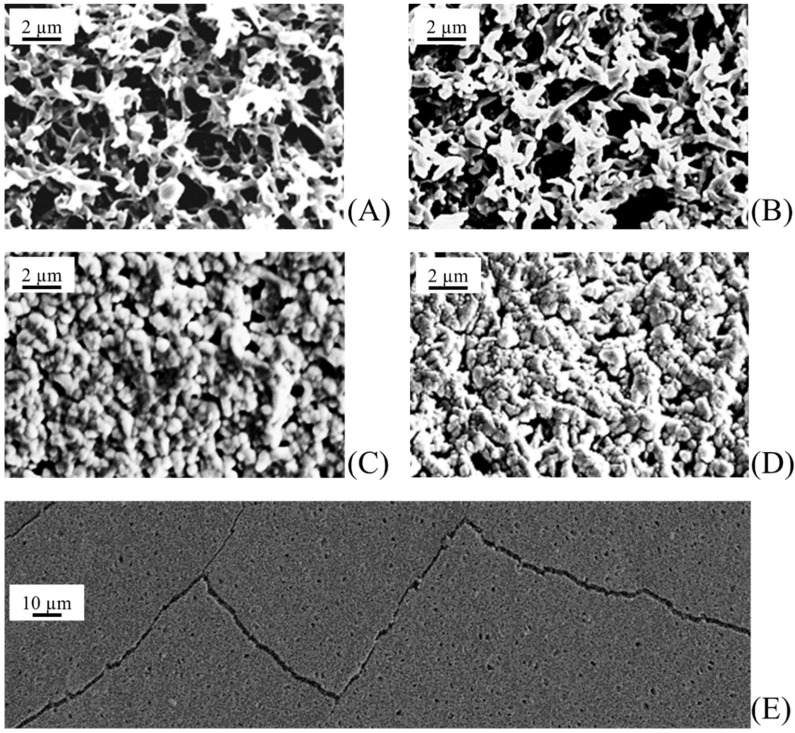
Morphology of virgin and TiO_2_ sputtered polyvinylidene difluoride (PVDF) membranes after different sputtering times (t): (**A**) virgin PVDF membrane; t = 0, (**B**) t = 1 h; (**C**) t = 2 h; (**D**) t = 3 h; (**E**) t = 4 h.

The different sputtering conditions (sputtering power and time, target distance, and gas pressure) were tested in order to find the optimal set of parameters able to give a homogeneous membrane coverage with no polymer substrate damage and very small photocatalyst nanoparticles. This last condition ensures a high photoactivity, with catalysis being a surface process. Due to inadequate sputtering conditions, typical sample drawbacks were inhomogeneous coverage, pore occlusion, and presence of cracks (damage of thin film), as shown in Figure 3. The best results—in terms of both coverage quality and nanoparticle size—were obtained with the sputtering conditions (sputtering power, target distance, gas pressure, sputtering time) shown in Table 1.

**Figure 4:**
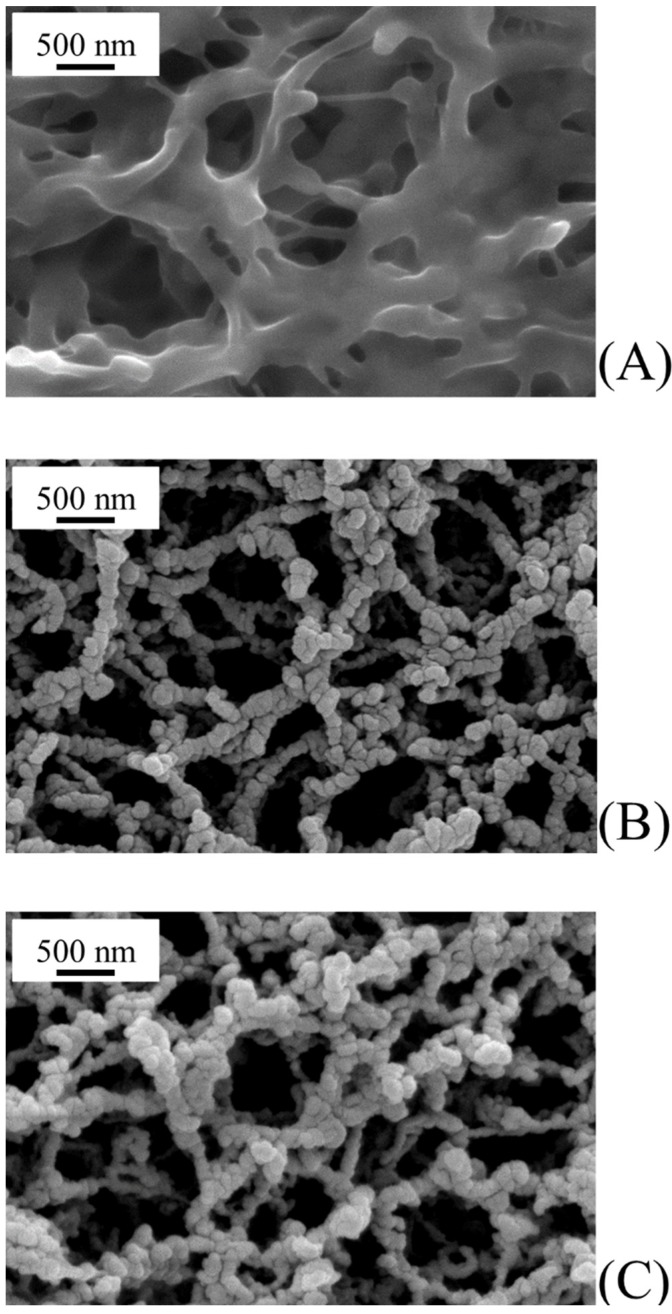
Morphology of virgin and sputtered PVDF membranes under the experimental conditions reported in Table 1: (**A**) virgin PVDF membrane; (**B**) ZnO sputtered PVDF membrane; and (**C**) TiO_2_ sputtered PVDF membrane.

Figure 4 shows the morphology of the top surface in virgin and sputtered PVDF membranes under the experimental conditions reported in Table 1. Both photocatalyst coatings (Figure 4B,C for ZnO and TiO_2_, respectively) were homogeneous with no evident alteration/damage of the virgin PVDF membrane (Figure 4A). In addition, no occlusion of membrane pores was present. Coatings had a cauliflower structure with aggregate diameters of around 100 nm and formed by agglomeration of smaller primary nanoparticles (see later).

**Figure 5:**
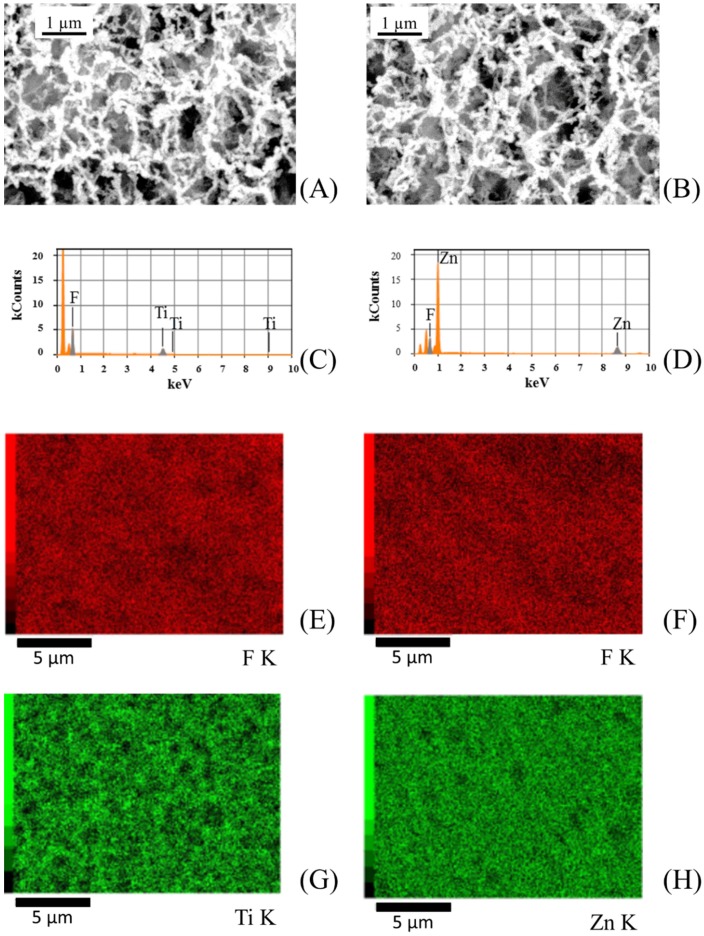
(**A**,**B**) Scanning electron microscopy (SEM) backscattering electron micrographs, (**C**,**D**) elemental mapping, and (**E**–**H**) energy-dispersive X-ray (EDX) analysis performed on TiO_2_ (on the **left**) and ZnO (on the **right**) sputtered PVDF membranes.

In order to further confirm that PVDF membranes were homogeneously covered with photocatalysts, SEM backscattering electron micrographs and spot EDX analysis on sputtered PVDF membranes were performed. Figure 5 shows SEM backscattering electron micrographs and EDX maps for ZnO and TiO_2_ sputtered membranes.

**Figure 6:**
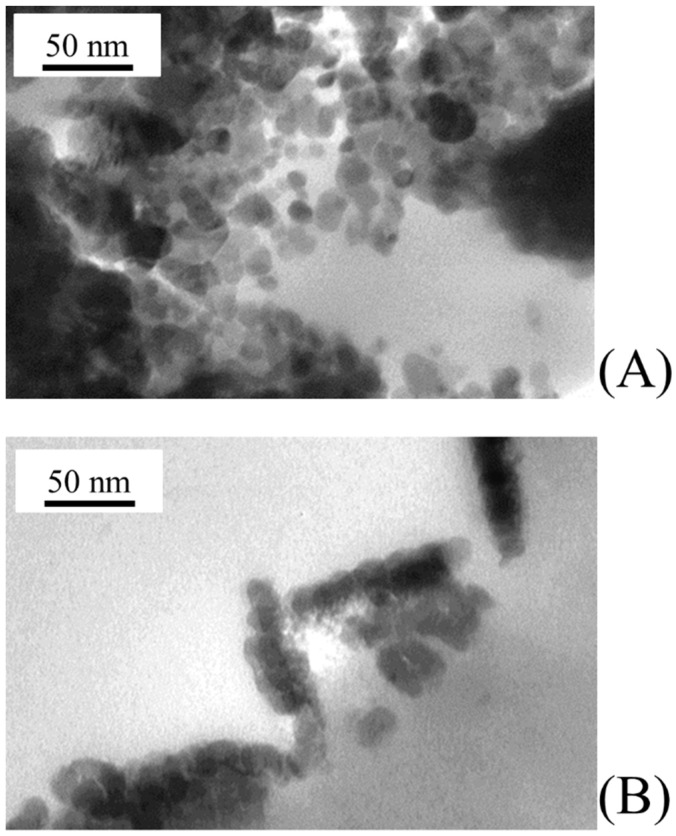
Morphology of catalyst nanoparticles grown on PVDF membranes under the experimental conditions reported in Table 1: (**A**) ZnO sputtered PVDF membrane; and (**B**) TiO_2_ sputtered PVDF membrane.

**Figure 7:**
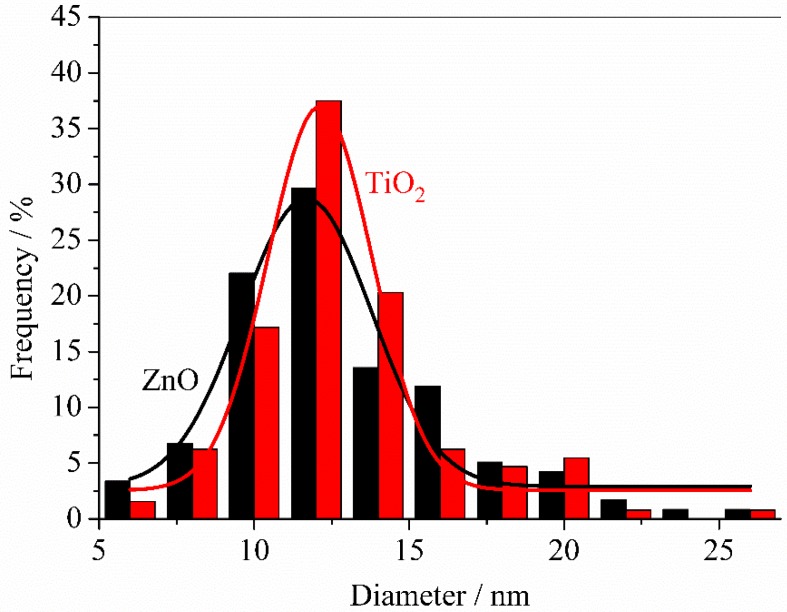
Distribution of diameters shown by primary nanoparticles present on ZnO and TiO_2_ sputtered PVDF membranes.

At larger magnifications, TEM analysis allows characterizing the morphology of primary nanoparticles grown on PVDF membranes. As shown in Figure 6A,B, both ZnO and TiO_2_ primary nanoparticles were rather spherical in shape with similar average diameters of 11.6 ± 4.2 and 12.1 ± 3.4 nm, respectively (Figure 7). Primary nanoparticles agglomerated into larger aggregates.

**Figure 8:**
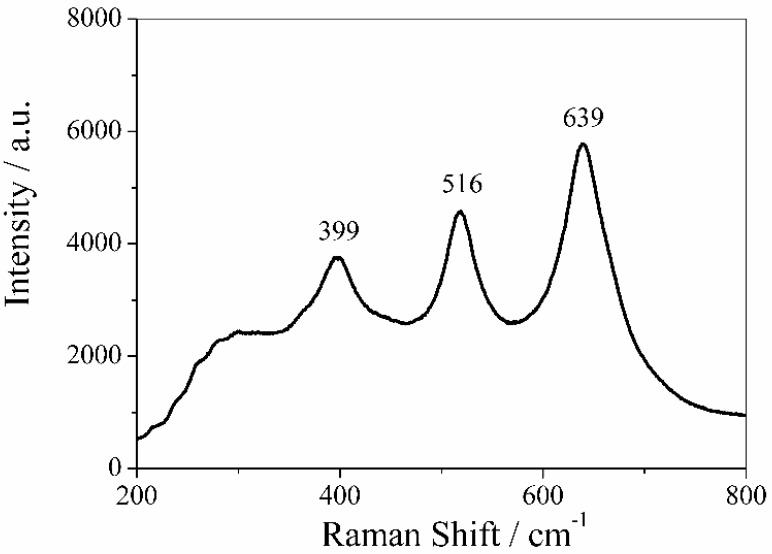
Raman spectrum of TiO_2_ nanoparticles sputtered on PVDF membrane. The peaks at 399, 516, and 639 cm^−1^ are associated to the Raman active modes B1g, A1g, and Eg, respectively, confirming the anatase structure of TiO_2_.

MicroRaman spectrum of PVDF membrane obtained by reactive sputtering using a Ti target is reported in Figure 8.

The Raman spectrum of TiO_2_ nanoparticles consisted of three peaks with strong intensities at 399, 516, and 639 cm^−1^, which can be associated to the Raman active modes B1g, A1g, and Eg of anatase structure of TiO_2_ thin layers. These values are in good agreement with the Raman bands reported in literature [43]. The fourth active Raman mode of anatase structure of TiO_2_, which is generally placed at 196 cm^−1^ (Eg mode), was out of the instrument range. It is expected that the deposition of ZnO and TiO_2_ thin films could change the hydrophilicity of virgin PVDF membranes.

Table 2 shows the static contact angles to pure water measured for virgin and sputtered membranes. Both ZnO and TiO_2_ thin films drastically reduced the contact angle, i.e., increased the hydrophilicity of virgin PVDF membrane from 61° to 27° and 26°, respectively, with a consequent decrease in fouling. It is important to recall that the photoactivity of ZnO and TiO_2_ layers is able to further overcome this drawback. Moreover, the increase in hydrophilicity is expected to have a positive effect in the membrane permeation properties. Table 2 also shows the transmembrane fluxes of virgin and sputtered membranes. The performant effect of catalyst thin film increased the transmembrane flux of virgin PVDF from a value of 200 to 760 and 710 L m^−2^ h^−1^ for ZnO and TiO_2_ thin films, respectively, as a result of the increased hydrophilicity. These results also confirm that the sputtered thin layer did not occlude the membrane pores.

In order to test their photocatalytic activity against pharmaceutical active compounds and organic pollutants, sputtered membranes were placed in a small continuous flow reactor where a water solution of either diclofenac sodium—a well-known anti-inflammatory drug—or methylene blue—a well-known organic dye—was circulated.

**Figure 9:**
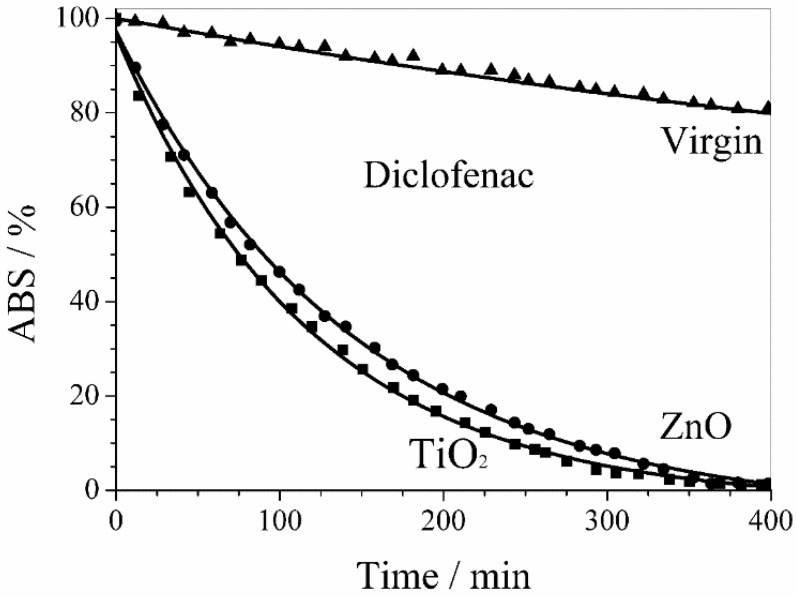
Photodegradation of diclofenac sodium salt by PVDF membranes with sputtered ZnO and TiO_2_ nanoparticles. The behavior of virgin PVDF membrane takes into account the UV photolysis of diclofenac.

Figure 9 shows the activity of ZnO and TiO_2_ sputtered membranes in the diclofenac sodium salt photodegradation. The drug photodegradation by PVDF membranes with sputtered ZnO and TiO_2_ nanoparticles followed a first order kinetics with similar rate constants of 6.8 × 10^−3^ min^−1^ and 8.3 × 10^−3^ min^−1^, respectively. An almost complete photodegradation of diclofenac sodium salt was obtained within 6 h in both cases.

**Figure 10:**
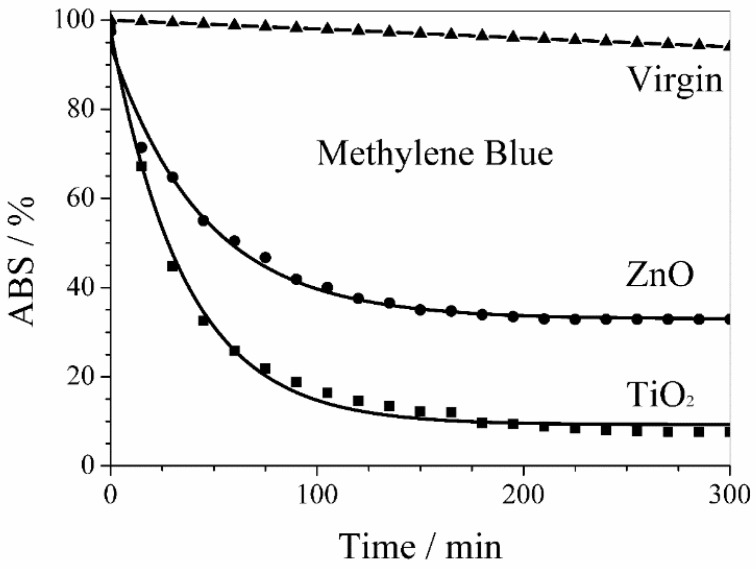
Photodegradation of methylene blue by PVDF membranes with sputtered ZnO and TiO_2_ nanoparticles. The behavior of virgin PVDF membrane takes into account the UV photolysis of methylene blue.

Similarly, Figure 10 shows the activity of ZnO and TiO_2_ sputtered membranes in the methylene blue photodegradation. Also in this case, the photodegradation kinetics of the organic pollutant by PVDF membranes with sputtered ZnO and TiO_2_ nanoparticles was a first order kinetics but with larger rate constants of 2.2 × 10^−2^ min^−1^ and 2.8 × 10^−2^ min^−1^, respectively. Nevertheless, the methylene blue photodegradation stopped after 4 h with a plateau of 33% and 8% for PVDF membranes with sputtered ZnO and TiO_2_ nanoparticles, respectively.

The behavior of virgin PVDF membrane reported in Figure 9 and Figure 10 takes into account the UV photolysis of diclofenac sodium and methylene blue, respectively.

Several parameters can affect the degradation efficiency of photocatalysts including the particular dye/drug, the pH of the solution, the presence of oxygen, the addition of hydrogen peroxide, the nanoparticle average size, and the amount and type of catalyst [44]. In particular, several experimental investigations have found that TiO_2_ nanoparticles show a photocatalytic efficiency higher than ZnO nanoparticles due to their band gap values [45,46]. Indeed, ZnO samples have a larger band gap, which leads to the production of less radicals and, consequently, to a lower dye photodegradation. On the contrary, TiO_2_ has a higher quantum yield engendered by a relatively slower electron-hole pair recombination, faster electron-hole pair migration to the surface, fewer defects, and exciton traps in the crystal lattice [47].

Even if kinetic rate constants are strongly dependent on membrane composition, investigated pollutants and the UV lamp power used [48], it is important to note that the obtained values of kinetic rate constants are the same order of magnitude as those found in other literature works where the photoactive nanomaterial was either directly synthesized or immobilized on polymer substrates [49,50,51]. These rate values make the CVD of photocatalyst nanoparticles on porous polymer membranes a suitable technique for applications in the field of advanced oxidation processes. In fact, the CVD of photocatalysts is a fast process that avoids expensive and time-consuming syntheses and cleaning post-treatments, as it is possible to directly sputter photocatalysts onto commercially available membranes while keeping good photocatalytic activities at the same time. Recycling of a catalyst is a very important property in practical applications. In order to assess the recycling properties of photocatalysts, ten photodegradation cycles were performed using the same film and fresh methylene blue. Both ZnO and TiO_2_ sputtered membranes were reused in successive runs without performing any cleaning procedure and gave percentages of degraded methylene blue similar to those obtained after the first run. These results demonstrated the ability of the ZnO and TiO_2_ sputtered membranes to fully preserve/restore their initial photocatalytic efficiency. In addition, the long-term stability of photocatalyst deposit onto polymer substrates was checked after ten cycles of successive photocatalysis processes. No evident damage was revealed in the nanoparticles layer morphology, confirming the main advantage of an easy reuse of membranes with sputtered catalysts over the homogeneous and heterogeneous catalysis processes, which suffer the drawbacks of catalyst recovery and damage of polymers used to functionalize membranes or bind catalysts [52]. Further work is in progress in order to control the primary particle size in a finer way.

## 4. Conclusions

In this work, the results of a CVD functionalization of polymer porous membranes with photocatalytic nanoparticles, and an application of their use in AOP were shown. The overall sputtering process took less than 1 h, which is lower than conventional times employed in organic synthesis processes of similar materials. The thickness of obtained surface coating by sputtered nanoparticles was found to depend on process conditions. The membranes functionalized with ZnO and TiO_2_ nanoparticles were characterized by contact angles lower than that shown by virgin membrane, making these composite membranes suitable for the filtration of aqueous solutions. The deposition of a thin layer of nanoparticles increased the transmembrane fluxes as hydrophilicity increased, and no pore occlusion occurred. In addition to its long-term stability and solvent-free features, the proposed process of membrane functionalization can be easily upscaled to manufacture membrane modules for the efficient degradation of organic pollutants generally found in wastewater.

## Figures and Tables

**Table 1:** Optimal sputtering parameters able to give a homogeneous coverage with no substrate damage and small nanoparticles.

Target	Photocatalyst Layer	Sputtering Power/W	Target Distance/10^−2^ m	Pressure/10^−6^ bar	Sputtering Time/min
ZnO	ZnO	35	8	P(Ar) = 4.5	30
Ti	TiO_2_	65	7	P(Ar) = 2.8 ^1^	60

^1^ P(O_2_) = 1.2 × 10^−6^ bar.

**Table 2:** Contact angle and transmembrane flux of virgin and sputtered PVDF membranes under the experimental conditions reported in Table 1.

Photocatalyst	Contact Angle/deg	Transmembrane Flux/L m^−2^ h^−1^
Virgin PVDF	61 ± 1	200 ± 15
ZnO	27 ± 2	760 ± 15
TiO_2_	26 ± 2	710 ± 15
